# An innovative automatic feeding device for preterm infants: promoting the development of sucking ability

**DOI:** 10.3389/fmedt.2025.1691199

**Published:** 2025-12-09

**Authors:** Fei Luo, Xiaoli Zhao, Yi Lin, Huiling Huang, Zinan Liu, Junhong Xu, Hongping Li

**Affiliations:** 1 Pediatrics Department, The Second Affiliated Hospital of Shantou University Medical College, Shantou, Guangdong, China; 2 Affiliated Shenzhen Children’s Hospital of Shantou University Medical College, Shenzhen, China; 3 Shenzhen Kaiyang Industrial Co., Ltd, Shenzhen, Guangdong, China

**Keywords:** automatic feeding device, preterm infants, enteral nutrition, gastric emptying, animal experiments

## Abstract

Preterm infants, particularly those born before 32 weeks of gestation, frequently face challenges in achieving full enteral nutrition due to underdeveloped sucking-swallowing-breathing coordination. Conventional feeding methods, such as the use of indwelling nasogastric tubes, overlook the importance of sucking activity, which is essential for the development of gastrointestinal motility and the secretion of digestive enzymes. To address this issue, we have developed a sucking-rewarded automatic feeding device specifically designed for preterm infants. The device features a specialized pacifier that detects sucking activity and triggers the delivery of a predetermined amount of milk into the stomach via a gastric tube. In addition to promoting sucking-induced satiety, the device continuously monitors sucking waveforms to assess infants’ viability and sucking maturity. In a clinical pilot study involving 25 preterm infants, those fed with the device demonstrated a significant increase in intestinal oxygen saturation compared with conventional gavage feeding (*p* < 0.05). Complementary experiments in 12 newborn beagle puppies showed faster gastric emptying rates (*p* < 0.01) and elevated gastrointestinal hormone levels (*p* < 0.05) when using the device. These findings highlight the clinical potential of the proposed device in improving feeding safety, efficiency, and developmental outcomes in preterm infants, and warrant further large-scale clinical trials to validate its long-term efficacy and integration into neonatal care.

## Introduction

The development and maturation of sucking behavior represent crucial milestones in the overall development of preterm infants. Sucking skills typically emerge at approximately 20 weeks of gestation, evolving from an immature pattern into a coordinated sucking-swallowing-breathing rhythm that is essential for effective oral feeding (1, 2). The neural regulation provided by the suck central pattern generator plays a vital role in this developmental process (3). Although sucking abilities naturally improve with advancing gestational age, targeted medical interventions can further facilitate this progression (4–6). Nonetheless, successful oral feeding is largely contingent upon the maturation of sucking-swallowing-breathing coordination, typically occurring around 34 weeks of gestation (7–9). Preterm infants born prior to this milestone are at an increased risk of aspiration during oral feeding, often necessitating reliance on gastric tube feeding.

Extended dependence on gastric tube feeding, in the absence of sufficient oral simulation, may impede the development of coordinated sucking-swallowing-breathing skills and result in persistent feeding difficulties and related complications (6, 10–13). In contrast, early initiation of sucking and oral stimulation has been associated with enhanced development of sucking abilities, increased secretion of gastrointestinal hormones, and improved sleep quality, all of which are essential for optimal gastrointestinal function and overall well-being (14–19). Conventional feeding methods typically involve the direct infusion of nutritional formula into the stomach at fixed intervals by nursing staff, a process that often fails to account for the infants’ hunger signals, despite evidence that such hunger signals can effectively promote the maturation of oral sucking function (20).

Previous studies have explored the development of monitoring systems designed to capture physiological parameters associated with oral feeding, thereby facilitating the evaluation of preterm infants’ feeding behavior (21–24), but they mostly focused on the preterm infants with >34 weeks of gestation and without automatic feeding. Building upon these efforts, our team has developed a novel sucking-rewarded automatic feeding device specifically targeted at preterm infants born before 34 weeks of gestation. This device is equipped with a special pacifier capable of sensing sucking activity and automatically delivering a predetermined volume of milk into the stomach upon detection of sucking activity. It is designed to reinforce sucking reflexes and enhance gastrointestinal function, as well as the coordination of sucking-swallowing-breathing, thereby promoting overall feeding success. Moreover, the device's capacity to analyze sucking data provides valuable insights into the maturation and vitality of an infant's sucking behavior, facilitating early detection and intervention for neurological and systemic complications.

## Materials and methods

### Design of automatic feeding device for preterm infants

**Figure 1:**
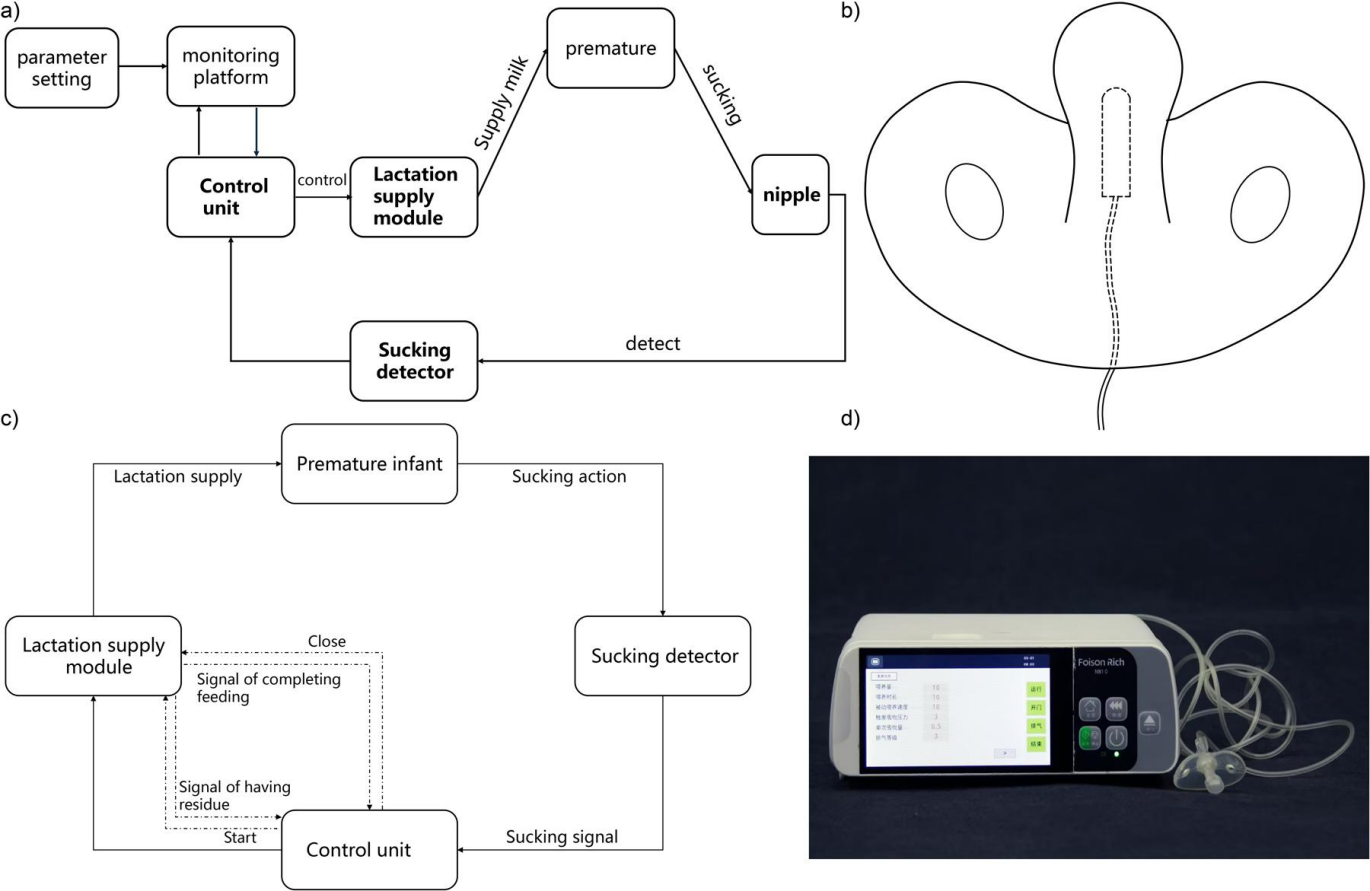
Illustration of the sucking-rewarded automatic feeding device. **(a)** Conceptual diagram of whole device, **(b)** Schematic diagram of the pacifier, **(c)** Operational flow of the feeding process, **(d)** Photograph of the assembled automatic feeding device.

The automatic feeding device (Patent No. PCT/CN2018/113541) was developed to synchronize milk delivery with the natural sucking behavior of preterm infants, thereby promoting physiologically appropriate feeding. As shown in Figure 1a, the device consists of four main components: a sucking detector, a control unit, a lactation supply module, and a stomach tube. And the device integrates an LCD interface and a control detection board, allowing real-time monitoring and intuitive adjustment of feeding parameters.

The pacifier-type detector (Figure 1b) identifies mechanical deformation generated during sucking and transmits corresponding pressure signals to the controller. The control unit then processes these signals and dynamically adjusts the motor speed of the lactation supply module, ensuring milk delivery consistent with the infant's sucking activity. Detailed technical specifications are provided in the Supplementary Methods.

### Operation of feeding process

The feeding process consists of four sequential stages (Figure 1c):

1. preparation stage: The lactation supply module is preloaded with the volume of milk determined by clinical guidelines.
2. active lactation stage: Upon detection of sucking signals, the control unit activates the lactation supply module to deliver small boluses of milk in response to each suck.
3. passive lactation stage: If residual milk remains after active feeding, the system automatically switches to a constant-rate mode to complete milk delivery.
4. rest stage: The infant rests for 2–3 h before the next feeding cycle.

This design ensures responsive and safe milk delivery while preventing passive overfeeding.

### Reference algorithm for milk pumping volume per suck

For preterm infants receiving approximately 150 mL/kg/day of milk at 3-hour intervals, each feeding corresponds to ∼18.75 mL/kg. Assuming a 15-minute active feeding session and a sucking frequency of 1 suck/s (300–600 suck cycles), the device delivers approximately 0.1–0.6 mL per suck, adjustable based on clinical tolerance (7). Calculation details are provided in Supplementary Methods.

### Clinical study subjects

A total of 25 neonates with a gestational age of less than 32 weeks admitted to the neonatal intensive care unit at Shenzhen Children's Hospital were enrolled. Participants were randomly assigned to either the automatic feeding device group (*n* = 13, experimental group) or the conventional gastric tube feeding group (*n* = 12, control group). This study was designed as a proof-of-concept trial to assess the feasibility, safety, and preliminary efficacy of the automatic feeding device. All preterm infants were fed either expressed breast milk from their mothers or preterm infant formula (for cases where breast milk was unavailable). Prior to administration, milk was warmed to approximately 37°C.

The study adhered to the Declaration of Helsinki and was approved by the Ethics Committee of Shenzhen Children's Hospital (No.202312302). Inclusion criteria were: (1) a gestational age less than 32 weeks; (2) Apgar scores of ≥8 at 1, 5, and 10 min post-delivery; and (3) absence of severe complications. Exclusion criteria included severe apnea, intrauterine growth retardation, serious infectious diseases, major congenital malformations, immune dysfunctions, or neurological disorders such as grade III–IV intraventricular hemorrhage or periventricular leukomalacia. Written informed consent was obtained from the parents or guardians.

### aEEG monitoring and intestinal oxygenation monitoring

Amplitude-integrated EEG (aEEG) recordings were performed using the Nicolet One system (Natus Medical, Pleasanton, CA, USA) within 48 h of admission and repeated on day 14 (D14) and day 28 (D28). Recordings followed the international 10–20 system and lasted 4–6 h under stable clinical conditions.

Intestinal oxygen saturation (SO₂) was assessed using a near-infrared spectroscopy (NIRS) system (EGOS-600A, Suzhou Aegean Biomedical, China). Measurements were obtained 30 min before feeding and up to 60 min after feeding completion, divided into T0 (during feeding), T30 (0–30 min post-feeding), and T60 (30–60 min post-feeding). Device calibration and probe placement procedures are detailed in Supplementary Methods.

### Animal experiments

All animal experiments complied with the Animal Ethical Committee of Shenzhen TopBiotech Co., Ltd., China. Beagle puppies were selected as experimental models due to their neonatal gastrointestinal motility and peristaltic mechanisms being comparable to those of human preterm infants (25). The study compared milk delivery efficiency and physiological responses between the automatic feeding device (experimental group, *n* = 6) and traditional syringe-based gastric feeding (control group, *n* = 6). Formula milk was reconstituted according to the manufacturer's instructions and maintained at approximately 37°C prior to administration.

Gastrointestinal hormones, including cholecystokinin, neurotensin, pancreatic polypeptide, ghrelin, gastric inhibitory polypeptide (GIP), gastrin, glucagon, and secretin were quantified using ELISA kits obtained from RabBiotech Life, Inc. Comprehensive mechanical setup and feeding control protocols are described in the Supplementary Methods.

### Statistical analysis

Continuous variables were analyzed using Student's *t*-test, and categorical variables using chi-square tests. Statistical analyses were performed using *R* software, with a significance level of *p* < 0.05.

## Results

### Characteristics of the automatic feeding device

This developed pacifier is engineered to enable infants to detect mechanical stress and deformation during sucking. The sucking force is transmitted through a dedicated sampling channel to a piezoresistive silicon pressure sensor, thereby generating electrical signals. This sensor was meticulously selected for its high consistency, rapid response, and stable signal output, qualities that render it highly suitable for the oral environment. In particular, a piezoresistive silicon pressure sensor equipped with an onboard ASIC was chosen, fully calibrated, and temperature compensated for various parameters, including:

Startup time (from power on to data ready): 2.8–7.3 ms

Response time: 0.46 ms

Accuracy: ± 0.25% FSS

Position sensitivity: ± 0.15% FSS

Total error band (TEB): ± 1% FSS

Long term stability (1,000 hours, 25 ℃): 0.5% FSS

The sensor converts variations in sucking force into electrical signals, which are subsequently transmitted to a microcontroller for processing. The measured pressure values are then compared with a predefined threshold to trigger the feeding mechanism. To ensure precise force monitoring, the pacifier's mechanical resilience and internal volume consistency were calibrated against a high-precision pressure gauge.

The lactation supply module integrates a motor and an associated speed monitoring circuit (Supplementary Figure S1). Under the control of the device's controller, the motor drives gears in the milk supply module to dispense a milk emulsion. A Hall sensor monitors the motor's rotational speed and turns, thereby determining both the volume of milk delivered and any residual emulsion. Additionally, a bubble detection sensor continuously monitors the presence of milk within the delivery conduit to ensure uninterrupted operation. The device is further equipped with an LCD panel and a CPU controlled calculation motherboard, which facilitate individualized feeding adjustments based on the varying sucking abilities of premature infants. The assembled automatic feeding device is shown in Figure 1d.

Clinicians determine the milk volume per feeding session and the corresponding pumping duration based on the infant's physical condition. The feeding strategy consists of two stages: an active milk supply stage and a passive milk supply stage, with the duration of each stage preset according to the infant's condition. During the active stage, when sucking signals are detected, the controller activates the milk supply module to deliver a small volume of milk (typically ranging from 0.1 to 0.6 mL per suck) into the preterm infant's stomach. This volume is adjustable according to clinical needs and the infant's tolerance. If the active feeding period elapses before complete milk delivery, the device automatically transitions to the passive stage, wherein the remaining milk is dispensed at a constant rate over a predetermined duration.

Throughout the active feeding stage, the device continuously monitors and analyzes oral sucking signals to assess both the maturity and variability of the sucking behavior. These real-time evaluations allow caregivers to adjust device parameters, thereby optimizing feeding and potentially serving as an early indicator of health issues. The integrated display panel provides real-time feedback on sucking activity and milk output, and the resulting sucking curve is used to evaluate the maturity of sucking behavior in premature infants based on a five-stage descriptive scale of infant nutritional sucking development (1). Overall, this feeding strategy combines dynamic monitoring, adjustable parameters, and real-time feedback to ensure optimal feeding and to support the assessment of sucking development in premature infants.

### Enhanced neurological development and intestinal oxygenation saturation in preterm infant feeding with the device

All participants are Han Chinese residing in Shenzhen city. No significant differences were observed between the two groups in terms of gestational age, mode of delivery, gender distribution, birth weight and birth length.

**Figure 2:**
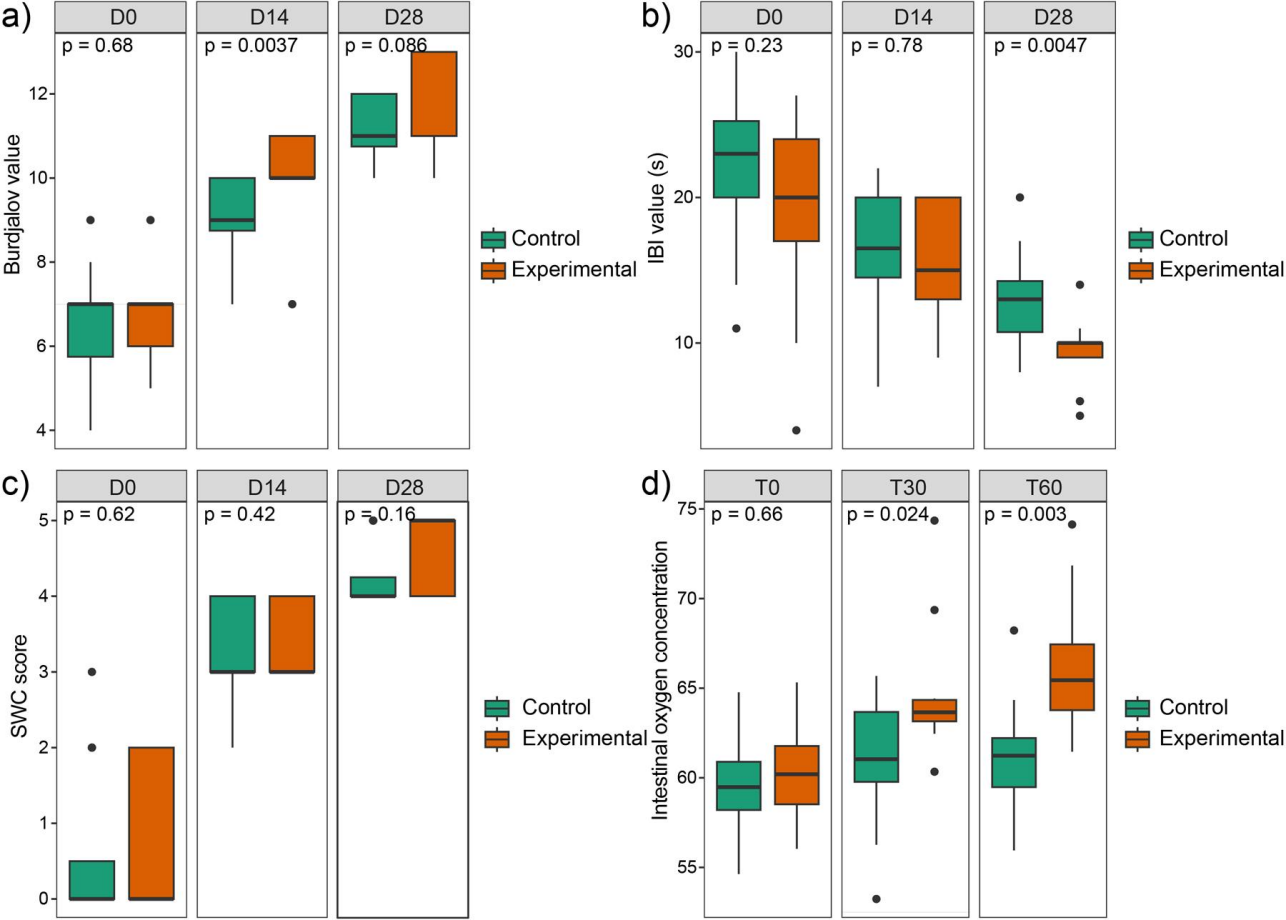
Comparisons of aEEG measurements and intestinal oxygenation saturation between the experimental and control groups. **(a)** The differences of Burdjalov score between the two groups. **(b)** The differences of IBI duration between the two groups. **(c)** The differences of SWC score between the two groups. **(d)** The differences of intestinal oxygenation saturation between the two groups.

Baseline aEEG measurement at day 0 (D0) showed no significant differences between the experimental and control groups across Burdjalov score, interburst interval (IBI) duration, and sleep-wake cycling score (SWC). After two weeks of intervention (D14), the experimental group exhibited significantly higher Burdjalov score than the control group (*P* < 0.05, Figure 2a), indicating improved brain maturation. At the completion of the 4-week intervention period (D28), although the experimental group continued to display higher Burdjalov score, the difference was not statistically significant. Notably, at D28, the IBI duration, an indicator of cortical activity development, was significantly improved in the experimental group compared to the control group (*P* < 0.05, Figure 2b). However, no significant differences in SWC score were observed between the groups throughout the study period (Figure 2c), suggesting comparable development of sleep-wake cycling patterns.

Regarding intestinal oxygen saturation, no significant differences were observed between the two groups at T0. In contrast, at both T30 and T60, the experimental group exhibited significantly higher intestinal oxygen saturation levels compared to the control group (*P* < 0.05, Figure 2d), indicating enhanced intestinal perfusion in response to the intervention.

### Accelerated gastric emptying in beagle puppies feeding with the device

**Figure 3:**
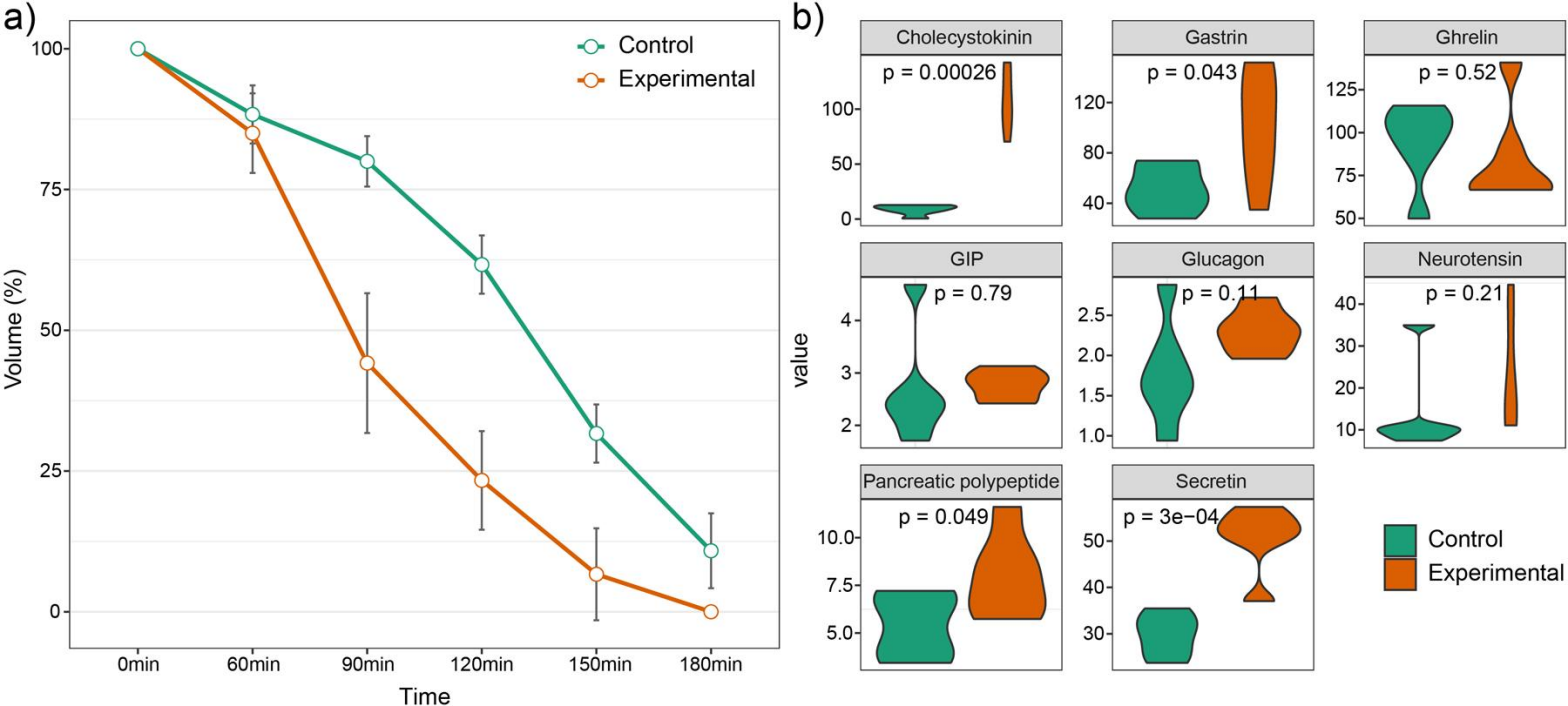
Comparisons of gastric emptying curve and gastrointestinal hormone levels in beagle puppies between the experimental and control groups. **(a)** The differences of gastric emptying curve between the two groups. **(b)** The differences of gastrointestinal hormone levels between the two groups.

Figure 3a presented the gastric emptying profiles for both the experimental group and the control group at various time points (0, 60, 90, 120, 150, and 180 min) following feeding. The results revealed a marked difference in gastric emptying between the two groups. Specifically, the experimental group achieved approximately 50% gastric emptying in about 80 min, a profile that closely resembles the oral feeding curve observed in full-term human newborns (26). In contrast, the control group required approximately 130 min to reach 50% gastric emptying. While no significant differences in gastric emptying were observed between the two groups at 0- and 60-minutes post-feeding (*P* > 0.05), significant differences emerged at 90, 120, 150, and 180 min (*P* < 0.05). These findings suggested that the automatic feeding device promotes more efficient gastric emptying, leading to a more rapid and complete clearance of gastric contents within the observed timeframe.

### Increased levels of gastrointestinal hormones in beagle puppies feeding with the device

To evaluate changes in gastrointestinal hormone levels in the experimental group, we measured the concentrations of cholecystokinin (ng/mL), neurotensin (ng/mL), pancreatic polypeptide (pg/mL), ghrelin (ng/mL), gastric inhibitory polypeptide (pg/mL), gastrin (ng/mL), glucagon (ng/mL), and secretin (ng/mL). The results demonstrated that the experimental group exhibited significantly higher levels of cholecystokinin (108.10 ± 27.87 vs. 8.54 ± 4.55, *P* < 0.01), gastrin (101.19 ± 44.66 vs. 51.89 ± 18.86, *P* = 0.043), pancreatic polypeptide (7.94 ± 2.19 vs. 5.38 ± 1.70, *P* = 0.049), and secretin (50.72 ± 7.16 vs. 25.19 ± 12.0, *P* < 0.01) compared with the control group (Figure 3b). These findings demonstrated that the automatic feeding device enhances the secretion of key gastrointestinal hormones.

## Discussion

This study introduced an automatic feeding device designed for premature infants requiring enteral nutrition via a gastric tube, offering a novel approach to feeding this vulnerable population. By detecting sucking signals within the oral cavity, the device delivers a predetermined amount of emulsion into the stomach through a controlled milk supply mechanism. Unlike traditional gastric tube feeding, this innovative method leverages the satiety reward mechanism triggered by the infant's sucking activity.

Continuous and dynamic monitoring of sucking signals is particularly crucial for premature infants, especially those born before 32 weeks of gestation, who often exhibit immature sucking patterns. By referencing the five stages of infant sucking development, this approach enables the assessment of whether a premature infant's sucking function aligns with their gestational age, serving as an early prognostic indicator (1, 8). Furthermore, adjusting milk volumes based on individual sucking ability allows for personalized feeding strategies, providing a reliable basis for transitioning to full oral feeding. Comparing an infant's sucking activity before and after intervention facilitates milk volume adjustments and may serve as an early diagnostic indicator for conditions that lacking characteristic symptoms, thereby aiding in early disease detection and intervention.

Preterm infants fed using this device exhibited higher Burdjalov score, shorter IBI duration and improved intestinal oxygenation. The increase in Burdjalov score and the reduction in IBI duration reflect advancing brain maturation and enhanced cortical function, which may reduce the risk of neurodevelopmental impairments and improve long-term prognostic outcomes (27, 28). Additionally, the observed increase in intestinal oxygenation saturation suggests improved splanchnic perfusion, which may lower the risk of necrotizing enterocolitis and feeding-related complications by enhancing oxygen delivery to the immature gut (29, 30). Improved intestinal oxygenation may also contribute to systemic oxygen balance, reducing hypoxic stress and stabilizing cerebral oxygenation, which is critical for neurodevelopment (29). However, the sample size was relatively small, which may limit the statistical power and generalizability of the findings. Future studies with larger sample sizes and extended follow-up periods are needed to confirm these findings.

To further assess the physiological effects of the device, we compared gastric emptying and gastrointestinal hormone levels in beagle puppies fed with the device vs. those receiving conventional gastric tube feeding. The results demonstrated significantly accelerated gastric emptying and increased secretion of gastrointestinal hormones in puppies fed with the device, with the gastric emptying curve closely resembling the physiological gastric emptying patterns (25). While these findings suggest that the device effectively promotes gastric emptying, further clinical studies are essential to evaluate its efficacy across preterm infants of varying gestational ages. Building upon these preliminary findings, future work will focus on validating the efficacy and safety of the device in larger, multicenter clinical trials involving diverse neonatal populations.

Patterned feeding techniques that incorporate tactile stimulation during feeding have been shown to significantly improve the achievement of full oral feeding at an earlier stage and contribute to long-term neurological development, including language and cognition function (31, 32). Unlike traditional gastric tube feeding, which bypasses oral sensory experiences, the novel feeding device in this study delivers milk to the stomach through suction, allowing premature infants to engage in oral tactile stimulation during feeding. However, the current design lacks taste and esophageal stimulation, which are essential factors in facilitating feeding progression (31, 33). Integrating olfactory and oral sensory stimulation, along with esophageal activation by food particles, may further enhance feeding efficiency and overall development in preterm infants.

Previous studies have demonstrated the efficacy of novel feeding interventions, such as micro-sterile water stimulation in the pharynx and middle esophageal, in improving feeding outcomes in newborns with swallowing difficulties. For example, Jadcherla et al. reported that this technique effectively stimulated and enhanced the maturation of the swallowing reflex, leading to a significantly higher success rate of oral feeding (20). Additionally, Choi et al. found that bolus temperature influences esophageal motor function, with a 45°C bolus significantly improving esophageal peristalsis (34). These findings highlight potential modifications for future iterations of the feeding device. Incorporating small amounts of sterile water or milk into the mouth and esophagus during feeding, with adjustments for temperature and taste stimulation, could promote the maturation of oral feeding skills. Moreover, equipping the device with pressure sensors along the stomach tube in the pharynx and esophagus could provide valuable data on swallowing pressure dynamics, enabling a more precise assessment of oral feeding readiness and intervention timing.

In summary, the novel feeding device designed for premature infants enhances sucking ability and promotes gastric emptying through sucking activity, providing an innovative and effective solution to feeding challenges in this population. Its ability to continuously and dynamically detect sucking activity enables clinicians to assess sucking maturity and monitor changes in premature infants’ physiological status more intuitively. This study contributes new insights into the development of individualized feeding strategies for premature infants. By leveraging the capabilities of this device, healthcare providers can tailor feeding regimens to meet the specific needs of each infant, ultimately improving clinical outcomes and supporting optimal neurodevelopment in this high-risk population.

## Data Availability

The original contributions presented in the study are included in the article/Supplementary Material, further inquiries can be directed to the corresponding author.

## References

[1] Lau C. Development of suck and swallow mechanisms in infants. Ann Nutr Metab. (2015) 66(Suppl 5):7–14. 10.1159/000381361 26226992 PMC4530609

[2] Indrio F Neu J Pettoello-Mantovani M Marchese F Martini S Salatto A Development of the gastrointestinal tract in newborns as a challenge for an appropriate nutrition: a narrative review. Nutrients. (2022) 14:1405. 10.3390/nu14071405 35406018 PMC9002905

[3] Barlow SM. Oral and respiratory control for preterm feeding. Curr Opin Otolaryngol Head Neck Surg. (2009) 17:179–86. 10.1097/MOO.0b013e32832b36fe 19369871 PMC2724868

[4] Czajkowska M Fonfara A Królak-Olejnik B Michnikowski M Gólczewski T. The impact of early therapeutic intervention on the central pattern generator in premature newborns - a preliminary study and literature review. Dev Period Med. (2019) 23:178–83. 10.34763/devperiodmed.20192303.178183 31654996 PMC8522407

[5] Pineda R Dewey K Jacobsen A Smith J. Non-nutritive sucking in the preterm infant. Am J Perinatol. (2019) 36:268–76. 10.1055/s-0038-1667289 30081403 PMC6561102

[6] Kamity R Kapavarapu PK Chandel A. Feeding problems and long-term outcomes in preterm infants-A systematic approach to evaluation and management. Children (Basel). (2021) 8:1158. 10.3390/children8121158 34943354 PMC8700416

[7] Gewolb IH Vice FL. Maturational changes in the rhythms, patterning, and coordination of respiration and swallow during feeding in preterm and term infants. Dev Med Child Neurol. (2006) 48:589–94. 10.1017/S001216220600123X 16780629

[8] Lau C. Development of infant oral feeding skills: what do we know? Am J Clin Nutr. (2016) 103:616S–21. 10.3945/ajcn.115.109603 26791183 PMC4733254

[9] Amaizu N Shulman R Schanler R Lau C. Maturation of oral feeding skills in preterm infants. Acta Paediatr. (2008) 97:61–7. 10.1111/j.1651-2227.2007.00548.x 18052999 PMC2289993

[10] Anderson E Gregoski MJ Gehle D Head WT Hardy KT Chapman A Severity of respiratory disease is correlated with time of first oral feeding and need for a gastrostomy tube at discharge in premature infants born at <30 weeks of gestation. Pediatr Pulmonol. (2022) 57:193–9. 10.1002/ppul.25713 34596360

[11] Pados BF Hill RR Yamasaki JT Litt JS Lee CS. Prevalence of problematic feeding in young children born prematurely: a meta-analysis. BMC Pediatr. (2021) 21:110. 10.1186/s12887-021-02574-7 33676453 PMC7936467

[12] Jadcherla SR Khot T Moore R Malkar M Gulati IK Slaughter JL. Feeding methods at discharge predict long-term feeding and neurodevelopmental outcomes in preterm infants referred for gastrostomy evaluation. J Pediatr. (2017) 181:125–130.e1. 10.1016/j.jpeds.2016.10.065 27939123 PMC5724518

[13] Pahsini K Marinschek S Khan Z Urlesberger B Scheer PJ Dunitz-Scheer M. Tube dependency as a result of prematurity. J Neonatal Perinatal Med. (2018) 11:311–6. 10.3233/NPM-1799 30010147

[14] Yisahak SF Boone KM Rausch J Keim SA. The timing and quality of sleep was associated with dietary quality and anthropometry in toddlers born preterm. Acta Paediatr. (2023) 112:1453–60. 10.1111/apa.16750 36905082 PMC11154188

[15] Say B Simsek GK Canpolat FE Oguz SS. Effects of pacifier use on transition time from gavage to breastfeeding in preterm infants: a randomized controlled trial. Breastfeed Med. (2018) 13:433–7. 10.1089/bfm.2018.0031 29912580

[16] Bapat R Gulati IK Jadcherla S. Impact of SIMPLE feeding quality improvement strategies on aerodigestive milestones and feeding outcomes in BPD infants. Hosp Pediatr. (2019) 9:859–66. 10.1542/hpeds.2018-0243 31658999

[17] Mohammed A-R Eid A-R Elzehery R Al-Harrass M Shouman B Nasef N. Effect of oropharyngeal administration of mother’s milk prior to gavage feeding on gastrin, motilin, secretin, and cholecystokinin hormones in preterm infants: a pilot crossover study. JPEN J Parenter Enteral Nutr. (2021) 45:777–83. 10.1002/jpen.1935 32458450

[18] Butler R Moore M Mindell JA. Pacifier use, finger sucking, and infant sleep. Behav Sleep Med. (2016) 14:615–23. 10.1080/15402002.2015.1048451 26548755

[19] Kumar D. Sleep as a modulator of human gastrointestinal motility. Gastroenterology. (1994) 107:1548–50. 10.1016/0016-5085(94)90563-0 7926520

[20] Jadcherla SR Peng J Moore R Saavedra J Shepherd E Fernandez S Impact of personalized feeding program in 100 NICU infants: pathophysiology-based approach for better outcomes. J Pediatr Gastroenterol Nutr. (2012) 54:62–70. 10.1097/MPG.0b013e3182288766 21694638 PMC3800145

[21] Chen C-T Wang L-Y Wang Y-L Lin B-S. Quantitative real-time assessment for feeding skill of preterm infants. J Med Syst. (2017) 41:95. 10.1007/s10916-017-0744-1 28478534

[22] Wang Y-L Kuo H-C Wang L-Y Ko M-J Lin B-S. Design of wireless multi-parameter monitoring system for oral feeding of premature infants. Med Biol Eng Comput. (2016) 54:1061–9. 10.1007/s11517-015-1400-x 26429347

[23] Wang YL Hung JS Wang LY Ko MJ Chou W Kuo HC Development of a wireless oral-feeding monitoring system for preterm infants. IEEE J Biomed Health Inform. (2015) 19:866–73. 10.1109/JBHI.2014.2335742 25014981

[24] Ecevit A Erdogan B Anuk Ince D Aksu M Unal S Turan Ö Determination of oral feeding skills in late preterm, early term, and full-term infants using the neonatal oral feeding monitor (NeoSAFE). Ital J Pediatr. (2025) 51:38. 10.1186/s13052-025-01867-2 39920842 PMC11806788

[25] Wyse CA McLellan J Dickie AM Sutton DGM Preston T Yam PS. A review of methods for assessment of the rate of gastric emptying in the dog and cat: 1898-2002. J Vet Intern Med. (2003) 17:609–21. 10.1111/j.1939-1676.2003.tb02491.x 14529126

[26] Camps G van Eijnatten EJM van Lieshout GAA Lambers TT Smeets PAM. Gastric emptying and intragastric behavior of breast milk and infant formula in lactating mothers. J Nutr. (2021) 151:3718–24. 10.1093/jn/nxab295 34590118 PMC8643590

[27] Vesoulis ZA Paul RA Mitchell TJ Wong C Inder TE Mathur AM. Normative amplitude-integrated EEG measures in preterm infants. J Perinatol. (2015) 35:428–33. 10.1038/jp.2014.225 25521561 PMC4447544

[28] Burdjalov VF Baumgart S Spitzer AR. Cerebral function monitoring: a new scoring system for the evaluation of brain maturation in neonates. Pediatrics. (2003) 112:855–61. 10.1542/peds.112.4.855 14523177

[29] Dotinga BM Mintzer JP Moore JE Hulscher JBF Bos AF Kooi EMW. Maturation of intestinal oxygenation: a review of mechanisms and clinical implications for preterm neonates. Front Pediatr. (2020) 8:354. 10.3389/fped.2020.00354 32719756 PMC7347753

[30] Kuik SJ Van Zoonen AGJF Bos AF Van Braeckel KNJA Hulscher JBF Kooi EMW. The effect of enteral bolus feeding on regional intestinal oxygen saturation in preterm infants is age-dependent: a longitudinal observational study. BMC Pediatr. (2019) 19:404. 10.1186/s12887-019-1805-z 31684920 PMC6827212

[31] Lessen BS. Effect of the premature infant oral motor intervention on feeding progression and length of stay in preterm infants. Adv Neonatal Care. (2011) 11:129–39. 10.1097/ANC.0b013e3182115a2a 21730902

[32] Pickler RH Wetzel PA Meinzen-Derr J Tubbs-Cooley HL Moore M. Patterned feeding experience for preterm infants: study protocol for a randomized controlled trial. Trials. (2015) 16:255. 10.1186/s13063-015-0781-3 26041365 PMC4460964

[33] Muelbert M Lin L Bloomfield FH Harding JE. Exposure to the smell and taste of milk to accelerate feeding in preterm infants. Cochrane Database Syst Rev. (2019) 7:CD013038. 10.1002/14651858.CD013038.pub2 31311064 PMC6634986

[34] Choi YJ Park MI Park SJ Moon W Kim SE Kwon HJ The effect of water bolus temperature on esophageal motor function as measured by high-resolution manometry. Neurogastroenterology Motil. (2014) 26:1628–34. 10.1111/nmo.12441 25307526

