# Supplementary material for: An innovative automatic feeding device for preterm infants: promoting the development of sucking ability
Source: Front Med Technol. 2025 Dec 9;7:1691199. doi: 10.3389/fmedt.2025.1691199 (PMC12722832; doi:10.3389/fmedt.2025.1691199)
Supplement: Supplementary file 1 [file Datasheet1.pdf]

Supplementary Materials for

An innovative automatic feeding device for preterm infants: promoting the development of sucking ability

Fei Luo[1#], *Xiaoli Zhao*[2#], *Yi Lin*[2#], *Huiling Huang*[2], *Zinan Liu*[2], *Junhong Xu*[3], *Hongping Li*[2*]

[1] Pediatrics Department, The Second Affiliated Hospital of Shantou University Medical College, Shantou, Guangdong, China 515063
[2] Affiliated Shenzhen Children's Hospital of Shantou University Medical College, Shenzhen, 518000, China
[3] Shenzhen Kaiyang Industrial Co., Ltd, Shenzhen, Guangdong, China 518000

* Corresponding author: Hongping Li, Sep 1984, female, MD, Email: hongping_li@126.com

Supplementary Methods

Device overview and feeding operation

The automatic feeding device (Patent No. PCT/CN2018/113541) was developed to synchronize milk delivery with the natural sucking behavior of preterm infants. The device comprises four core components: a sucking detector, a control unit, a lactation supply module, and a stomach tube. One end of the stomach tube is positioned within the infant's stomach, while the other connects to the lactation supply module. The sucking detector, embedded within a specially designed pacifier, captures the mechanical stress and deformation generated during sucking. These mechanical signals are converted into electrical signals by a pressure sensor and transmitted to the control unit, which subsequently regulates the motor speed of the lactation supply module to ensure precise milk delivery into the stomach.

Feeding operation proceeds through four sequential stages:

1. Preparation phase: The lactation supply module is preloaded with a prescribed milk volume in accordance with clinical feeding protocols.
2. Active lactation phase: Upon detecting a sucking signal, the control unit activates the lactation supply module to dispense a preset amount of milk through the gastric tube. Continuous sucking reinitiates the process, maintaining synchronization between the infant's natural feeding rhythm and milk delivery.
3. Passive lactation phase: Once the stored milk has been fully dispensed, the lactation supply module signals the control unit, which then deactivates the system to prevent passive feeding. Alternatively, if residual milk remains after the active feeding stage, the control unit directs the lactation supply module to distribute the remaining milk uniformly through the stomach tube. The module then ceases operation once all residual milk is dispensed, thereby preventing further passive feeding.
4. Rest phase: After feeding completion, the infant enters a resting period of approximately 2–3 hours before the next feeding cycle.

This closed-loop control design allows the device to respond dynamically to each sucking event, providing a physiologically appropriate feeding pattern for preterm infants.

An integrated LCD display and control board enable real-time monitoring of flow rate, pressure, and operational status. Together, these components create a responsive and adaptive feeding system designed to emulate physiological milk delivery.

Sucking pressure detection module

The sucking pressure detection module employs a pressure-monitoring configuration in which the pacifier's deformation is transmitted via a sealed fluid conduit to the pressure acquisition port of the control unit. The conduit utilizes standard medical-grade connectors to ensure complete airtightness. Continuous deformation of the pacifier generates gas pressure variations that are converted into analog electrical

signals through a precision pressure sensor and displayed in real time on an LCD interface.
Signal conversion and computation are performed by a high-speed 32-bit microcontroller operating at a sampling rate of 10 kHz—significantly exceeding the 50-Hz recognition threshold of the human eye—allowing instantaneous translation of sucking activity into waveform amplitude changes. PressFure calibration is conducted against a certified high-precision reference instrument to ensure quantitative accuracy. A real-time monitoring platform further supervises the sucking signal and system output, allowing fine-tuned parameter adjustments under clinical supervision.

Lactation supply module

The dedicated lactation supply module ensures smooth, safe, and fully controllable milk delivery (Figure S1). The key components of the module include:

1. Buffer plate: Provides elastic deformation space for the milk conduit, enhancing flexibility and preventing structural stress.
2. Motor and Hall speed monitor: Function as the primary power source by driving the peristaltic plate along a predefined trajectory, ensuring a consistent milk flow into the infant's stomach.
3. Catheter fixing slot: Multiple dedicated slots are incorporated to secure the milk conduit in position, minimizing the risk of dislodgment or breakage.
4. Fixed support: Offers additional structural stability to the overall system.
5. Peristaltic tablet: Composed of a flexible polymer film coupled to a rotation axis, this plate translates motor motion into peristaltic compression of the conduit.
6. Pressure detector: Provides continuous feedback to identify blockages or flow interruptions in real time.
8. Liquid stop clamp: Prevents unintended milk flow when the motor is inactive.
9. Pipeline inspection: Dedicated to routine inspection and maintenance of the milk supply.
11. Bubble detector: Monitors for air bubbles within the milk conduit to prevent flow disruption and ensure safe feeding.

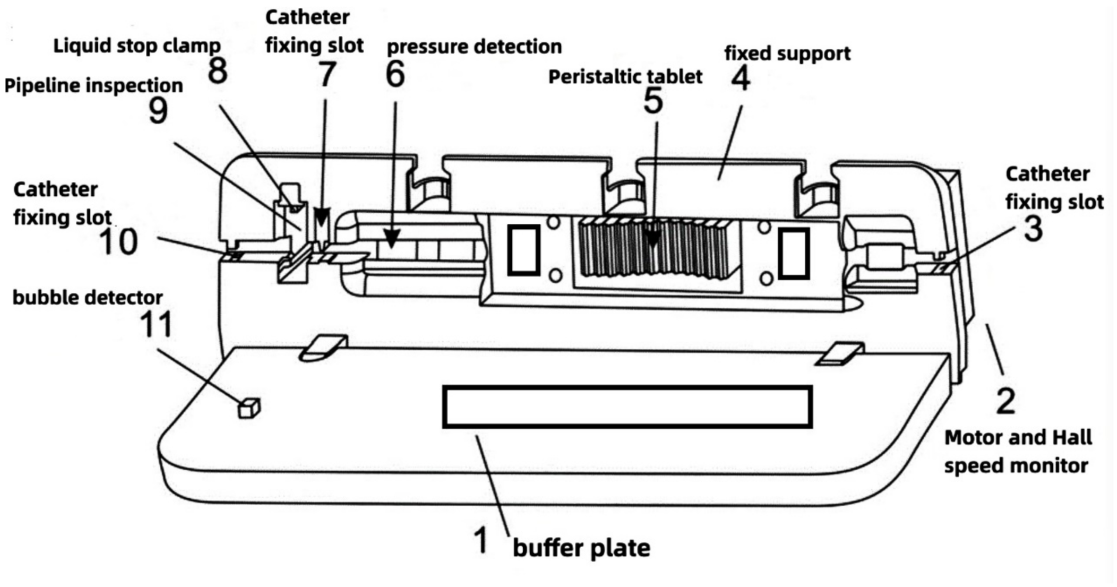

Figure S1. Schematic diagram of milk supply device: 1 buffer plate, 2 motor and Hall speed monitoring, 3-7-10 catheter fixing slot, 4 fixed support, 5 peristaltic tablet, 6 pressure detection, 8 liquid stop clamp, 9 pipeline inspection, 11 bubble detector.

Reference algorithm for milk delivery calibration

For premature infants with a prescribed daily milk intake of approximately 150 mL kg^{-1}, administered every 3 hours (equivalent to 18.75 mL kg^{-1} per feeding), the reference parameters for milk delivery were estimated as follows. Assuming an active feeding period of 15 minutes and an average sucking frequency of one suck per second—excluding brief pauses for breathing—each session consists of roughly 300–600 sucking events.

Given the system's 3-second response time per suck, effective activation occurs once every four sucks. Accordingly, the estimated milk volume delivered per suck ranges from 0.12 to 0.24 mL kg^{-1}, calculated as 18.75 mL kg^{-1} ÷ 600 × 4 (lower bound) to 18.75 mL kg^{-1} ÷ 300 × 4 (upper bound). For preterm infants with immature sucking ability, an effective delivery range of 0.1–0.6 mL per suck is considered appropriate, subject to clinical adjustment based on empirical data.

Amplitude-integrated electroencephalography (aEEG) monitoring

Amplitude-integrated electroencephalography (aEEG) recordings were obtained using a Nicolet One cerebral function monitor (Natus Medical Inc., Pleasanton, CA, USA). Baseline aEEG (D0) was recorded within 48 hours of admission, followed by subsequent recordings on days 14 (D14) and 28 (D28) to assess temporal changes in cerebral function during the intervention.

For each session, hydrogel electrodes were positioned in accordance with the international 10-20 system, employing a primary montage consisting of C3-P3 for the left hemisphere and C4-P4 for the right hemisphere, with Fz serving as the reference electrode. The Nicolet One system continuously recorded and filtered the raw EEG

signal (2-15 Hz), after which the signal was rectified, smoothed, and amplitude-integrated to produce a time-compressed aEEG tracing at a scale of 6 cm per hour. Recordings were conducted for a minimum duration of 4 hours (typically 4-6 hours) to capture representative brain activity patterns across various vigilance states. The artifact rejection algorithm was utilized to minimize interference from non-cerebral electrical activity. Measurements were performed under clinically stable conditions, avoiding feeding and procedural interference.

Intestinal oxygenation saturation monitoring

Intestinal oxygen saturation (SO_2) was continuously measured using the EGOS-600A near-infrared spectroscopy (NIRS) monitoring system (Suzhou Aegean Biomedical Electronics Co., Ltd., Suzhou, China). Prior to each measurement session, the NIRS device was calibrated according to the manufacturer's standard protocol. Infants were placed in a supine position for all measurements, and a sensor probe (detection depth: 1.5-2.5 cm) was positioned 0.5-1.0 cm below the umbilicus. The probe was secured to the measurement site using 3M adhesive film to ensure firm skin contact while preventing light leakage that could compromise measurement accuracy.

Continuous SO_2 monitoring commenced 30 minutes before feeding and continued until 60 minutes after feeding completion, with the NIRS system recording SO_2 values every 2 seconds. For analytical purposes, the monitoring period was divided into three-intervals: T0 (during feeding), T30 (0-30 min post-feeding), and T60 (30-60 min post-feeding). Standard clinical care was maintained for all subjects throughout the study period, and clinical staff were blinded to the NIRS monitoring results to prevent bias in clinical management. Data collectors received specialized training in proper probe placement and device operation to ensure the accuracy of intestinal oxygen saturation measurements.

Animal Experiments

All animal experiments were conducted in accordance with the guidelines established by the Animal Ethical Committee (Shenzhen TopBiotech Co., Ltd., China). The equipment utilized in this study was specifically designed to deliver enteral nutrition to premature infants by activating machine operation through pacifier sucking. Consequently, large mammalian cubs were selected as experimental subjects. Beagle puppies were chosen because their gastrointestinal anatomy and peristaltic function during the neonatal period closely resemble those of human preterm infants, making them an appropriate model for studying enteral feeding and digestive physiology. The primary objective was to compare the efficacy of an automatic feeding instrument with that of conventional gavage feeding using a syringe gastric tube.

Formulated milk powder was prepared following the manufacturer’s instructions. Seven-day-old male Beagle puppies with comparable body weights were randomly

assigned to either an experimental group (n = 6) and a control group (n = 6). The experimental group received milk feeding via the automatic device, while the control group was fed using traditional gastric tube feeding with a syringe. After 7 days of feeding, gastric emptying was evaluated, and blood samples were collected for gastrointestinal hormones analysis. Each feeding session was standardized at 40 mL per puppy, comprising 10 mL of iodohexanol and 30 mL of prepared milk. X-ray examinations were performed immediately after feeding and at 60, 90, 120, 150 and 180 minutes post-feeding. All puppies were fasted prior to imaging to ensure complete gastrointestinal emptying. Gastrointestinal hormones, including cholecystokinin, neurotensin, pancreatic polypeptide, ghrelin, gastric inhibitory polypeptide (GIP), gastrin, glucagon, and secretin were quantified using ELISA kits obtained from RabBiotech Life, Inc.

Gastric contents were assessed semi-quantitatively using a five-stage stratification system based on X-ray images compared with photographs taken at the end of feeding. The scoring criteria were as follows:

1 point: Nearly opaque with gastric filling between 75% and 100%

2 points: More than half full (gastric filling >50% to 75%)

3 points: Half filled (gastric filling >25% to 50%)

4 points: Partially filled (gastric filling >10% to 25%)

5 points: Minimal residual contents (gastric filling ≤10%)

To minimize observer bias, each radiograph was independently evaluated and scored by five radiology professionals. Gastric emptying scores were recorded at each time point for both experimental and control groups.

Following feeding, the puppies were placed in a warm, dry cardboard enclosure equipped with a soft cushion to ensure comfort without excessive restraint. A 5-day acclimatization period was implemented to minimize stress. Familiar caretakers were present during X-ray imaging, which was conducted in a left-side position at predetermined time points to further reduce discomfort.
